# Recent Advances in Carbon-Based Sensors for Food and Medical Packaging Under Transit: A Focus on Humidity, Temperature, Mechanical, and Multifunctional Sensing Technologies—A Systematic Review

**DOI:** 10.3390/ma18081862

**Published:** 2025-04-18

**Authors:** Siting Guo, Iza Radecka, Ahmed M. Eissa, Evgeni Ivanov, Zlatka Stoeva, Fideline Tchuenbou-Magaia

**Affiliations:** 1 Centre for Engineering Innovation and Research, School of Engineering, Computing and Mathematical Sciences, Faculty of Science and Engineering, University of Wolverhampton, Wolverhampton WV1 1LY, UK; s.guo3@wlv.ac.uk; 2 Research Institute of Healthcare Sciences, School of Pharmacy & Life Sciences, Faculty of Science and Engineering, University of Wolverhampton, Wulfruna Street, Wolverhampton WV1 1LY, UK; a.m.eissa@wlv.ac.uk; 3 Open Laboratory on Experimental Micro and Nano Mechanics (OLEM), Institute of Mechanics, Bulgarian Academy of Sciences, Acad. G. Bonchev Str. Block 4, 1113 Sofia, Bulgaria; ivanov_evgeni@imbm.bas.bg; 4 Research and Development of Nanomaterials and Nanotechnologies—NanoTech Lab Ltd., Acad. G. Bonchev Str. Block 4, 1113 Sofia, Bulgaria; 5 DZP Technologies Limited, Cambridge CB4 2HY, UK

**Keywords:** carbon-based sensors, humidity sensors, temperature sensors, mechanical sensors, multifunctional sensors, smart food packaging, smart pharmaceutical product packaging, real-time monitoring

## Abstract

All carbon-based sensors play a critical role in ensuring the sustainability of smart packaging while enabling real-time monitoring of parameters such as humidity, temperature, pressure, and strain during transit. This systematic review covers the literature between 2013 and 16 November 2024 in the Scopus, Web of Science, IEEE Xplore, and Wiley databases, focusing on carbon-based sensor materials, structural design, and fabrication technologies that contribute to maximizing the sensor performance and scalability with particular emphasis on food and pharmaceutical product packaging applications. After being subjected to the inclusion and exclusion criteria, 164 studies were included in this review. The results show that most humidity sensors are made using graphene oxide (GO), though there is some progress toward cellulose and cellulose-based materials. Graphene and carbon nanotubes (CNTs) are predominant in temperature and mechanical sensors. The application of composites with structural design (e.g., porous and 3D structures) significantly improves sensitivity, long-term stability, and multifunctionality, whereas manufacturing methods such as spray coating and 3D printing further drive production scalability. The transition from metal to carbon-based electrodes could also reduce the cost. However, the scalability, long-term stability, and real-world validation remain challenges to be addressed. Future research should further enhance the performance and scalability of carbon-based sensors through low-energy fabrication techniques and the development of sustainable advanced materials to provide solutions for practical applications in dynamic transportation environments.

## 1. Introduction

**Figure 1:**
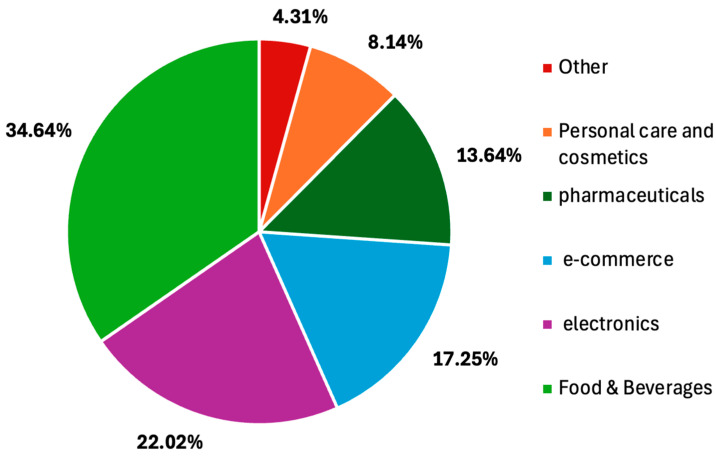
Global smart packaging market share 2023 (adapted from Fortune business insights, 2024) [8].

The rapid expansion of global trade and online shopping is coupled with an increased challenge in maintaining product quality, safety, and integrity during storage and transportation. Packaging plays an important role in maintaining product characteristics, especially for food and medical or pharmaceutical products, which are susceptible to contamination, degradation, spoilage, and physical damage leading to health risks, economic losses, and environmental impacts [1,2,3,4]. Indeed, GBP 14 billion of food is wasted annually in the UK [5], and the UK Health Security Agency, UKHSA (2022), reported GBP 5.7 million worth of vaccines wasted in 2019 and that 76% of these losses could have been prevented with better control of logistics, transportation, and storage conditions. At a global level, an estimated 1.3 billion tons of food are wasted throughout the food supply chain [6]. This waste corresponds to approximately 3.3 gigatons of CO_2_ equivalent emissions each year [7], accounting for 8–10% of total global greenhouse gas emissions [2]. If it were considered as a country, food waste would represent the third biggest source of these emissions worldwide [6]. These statistics underscore the urgent need for advanced packaging solutions to ensure product safety, minimize waste, and promote sustainability. This need is already exemplified by the expected smart packaging market growth from USD 23.33 billion in 2023 to USD 40.02 billion by 2032 [8], with food/beverages having the largest market share (34.6% share) when compared to other segments (Figure 1).

Smart packaging encompasses a range of technologies that integrate embedded sensors, identifiers, and various tools to enhance products safety, efficiency, sustainability, traceability, and user experience. This includes intelligent packaging, which monitors and communicates information about the product quality and state [9], and connected packaging through the Internet of Things and cloud systems [10]. This review focuses on sensors, which allow the monitoring and response of smart packaging to the dynamic transportation environments, where products are exposed to various fluctuating conditions such as humidity, temperature, and mechanical stress, thereby compromising their quality, safety, and integrity.

Packaging for food and medical/pharmaceutical products is designed to accommodate different internal pressures based on the packaging type, such as vacuum-seal packaging (0.1–100 kPa) [11], modified atmosphere packaging (5–50 kPa) [12], flexible or semi-rigid packaging (0.1–50 kPa) [13], and bulk packaging (1–500 kPa) [14]. Additionally, packaging is likely to encounter various mechanical stresses, including vibrations, compression and impacts from drops during storage, handling, and transportation (Table 1). These mechanical stresses can be monitored using mainly pressure and strain sensors. Pressure sensors measure the force per unit area of the material, helping to detect internal pressure changes to identify product leakage, packaging sealing damage, or defects, such as in blisters incomplete sealing and delamination. They also monitor external forces for preventing bursts and supporting the monitoring of packaging integrity under transportation stresses such as stacking, handling, and impacts. Conversely, strain sensors detect the deformation of the material [15] and can find applications in detecting cracks, fatigue, or tears that may jeopardize the packaging barrier properties and compromise the product safety, quality, and economical value.

Humidity sensors play vital roles in maintaining optimal moisture levels, issuing real-time alerts to prevent microbial growth and dehydration that could compromise product texture, potency, or freshness. Similarly, temperature sensors monitor thermal conditions, providing immediate feedback to prevent spoilage, protein denaturation, loss of functionality, and degradation. Multifunctional sensors integrate multiple detection capabilities, such as humidity, temperature, and mechanical sensing, into single platforms.

While acknowledging the key role of smart packaging in ensuring products quality and safety during transportation, reducing waste, extending shelf life and improving supply chain management [10], their general adoption is hindered by challenges related to cost, scalability, and environmental sustainability.

Recent advancements in sensor technology, particularly using carbon-based materials, offer a pathway to overcoming these challenges. These materials including graphene (G), graphene oxide (GO), reduced graphene oxide (rGO), carbon nanotubes (CNTs), carbon nanohorns (CNHs), graphene quantum dots (GQDs), and carbon black (CB), which exhibit exceptional properties, such as high electrical conductivity, mechanical strength and flexibility, thermal stability, light weight, and large surface area [25]. These properties enable enhanced sensing performance, miniaturization, and adaptability to diverse stimuli, including humidity, temperature, pressure, strain, and volatile organic compounds (VOCs). In addition, carbon-based materials are generally more cost-effective and relatively environmentally friendly compared to traditional high-performance materials such as silver [26]. However, certain high-purity carbon-based material production processes, particularly chemical vapor deposition (CVD), involve metal catalysts (nickel, iron, cobalt) which may cause heavy metal contamination due to residual catalyst particles [27]. Effective purification methods, including acid treatments, oxidation, thermal annealing, and magnetic separation, as well as employing low-toxicity or metal-free catalysts, have been developed to remove residual metal catalysts from carbon-based materials [27,28]. Technologies proposing catalyst-free synthesis include mechanical exfoliation, liquid-phase exfoliation, electrochemical exfoliation, Hummers’ process, plasma-enhanced catalyst-free CVD, and laser-induced methods [28,29,30,31] or the use of biomass-derived carbons sources [32], thus, offering environmentally friendly options. These technologies nevertheless still face challenges regarding mass production and production efficiency. To fully harness the environmental and economic potential of carbon-based materials, continued research is essential in developing greener synthesis techniques, effective catalyst management, and comprehensive lifecycle assessments. Although limited study has been performed on the interaction of carbon-based material such as graphene with the environment, it has been suggested that they can be biodegradable [33], therefore, offering the possibility of end-of-life disposal without the need for complex separation and recycling processes, especially when used in composites with other biodegradable polymers such as cellulose-based materials and polylactic acids.

While existing literature has reviewed carbon-based sensors, these reviews have typically focused on single-sensor functionalities (e.g., humidity, temperature, or mechanical sensors) and broadly addressed diverse applications ranging from environmental monitoring to healthcare wearables [34,35,36,37,38,39,40]. Existing reviews lack detailed discussions on novel carbon-based composite materials and advanced fabrication techniques developed specifically for packaging in relation to transportation conditions or transit. Equality, to the best of our knowledge, no previous review has simultaneously examined multiple sensing functionalities tailored to smart packaging for food and medical/pharmaceutical products. Furthermore, recent regulatory changes [41,42,43] coupled with technological advancements, alongside the growth of online shopping, have heightened the demand for specialized reviews on these applications to ensure compliance and efficacy. Consequently, there is a clear gap in the literature and this review significantly contributes to filling that by providing the first comprehensive and systematic analysis of recent advancements (2013–2024) in carbon-based humidity, temperature, mechanical, and multifunctional sensors for smart packaging applications for food and medical/pharmaceutical products that meet the acceptable conditions for safe storage and transportation (Table 1). Our review uniquely highlights the innovations in composite material design, structural optimization, and fabrication techniques that could enhance sensor performance specifically in packaging scenarios, offering new insights that bridge laboratory innovation with practical industrial scalability and regulatory compliance. Finally, the review identifies current research gaps and outlines potential future directions for cost-effective, scalable, and environmentally friendly on-package carbon-based sensors development in the food and pharmaceutical sectors.

## 2. Materials and Methods

The methodology for this review follows the guidelines of the Preferred Reporting Items for Systematic Reviews and Meta-Analyses (PRISMA) and the Prisma Checklist can be found in Supplementary Materials, Table S1. This offers a comprehensive review to ensure a comprehensive and focused analysis of recent advancements in carbon-based sensors [44]. It was registered in the Open Science Framework (https://doi.org/10.17605/OSF.IO/DYX4H, accessed on 2 April 2025).

### 2.1. Database Selection and Search Strategy

A comprehensive search was conducted across major academic databases, including Scopus, Web of Science, IEEE Xplore, and Wiley to ensure a broad coverage of relevant studies. The search strings were used to capture articles in all databases using a structured combination of keywords such as TITLE-ABS-KEY (‘carbon-based’ OR ‘graphene-based’ OR ‘graphene’ OR ‘carbon nanotube’ OR ‘graphene oxide’ OR ‘CNT’ OR ‘carbon’) AND TITLE-ABS-KEY (‘temperature sensor’ OR ‘humidity sensor’ OR ‘mechanical sensor’) AND TITLE-ABS-KEY (packaging AND food OR medical) AND PUBYEAR > 2013 AND PUBYEAR < 2025 AND (LIMIT-TO (DOCTYPE, ‘ar’)) AND (LIMIT-TO (LANGUAGE, ‘English’)).

### 2.2. Screening

After initial searching, a multi-step screening process was conducted. Firstly, after removing duplicates, each study was screened by titles and abstracts to access their potential relevance based on predefined inclusion and exclusion criteria (Table 2).

Articles that met the inclusion criteria underwent a detailed full-text review to ensure alignment with the research objectives. To ensure comprehensiveness, citation screening was employed during the full-text review. References within selected articles were assessed to identify additional relevant articles not captured in the database search. Newly identified articles were subjected to the same screening and inclusion criteria.

### 2.3. Data Extraction

Data extraction was conducted by S.G. using Microsoft Excel for data synthesis and presentation. For each study, the critical information like carbon-based material type, sensor design, fabrication methods, and performance metrics (e.g., sensitivity, response time, operating range, and durability) were recorded and compiled into tables. Owing to the heterogeneity of study designs, only a qualitative assessment was employed.

## 3. Results

**Figure 2:**
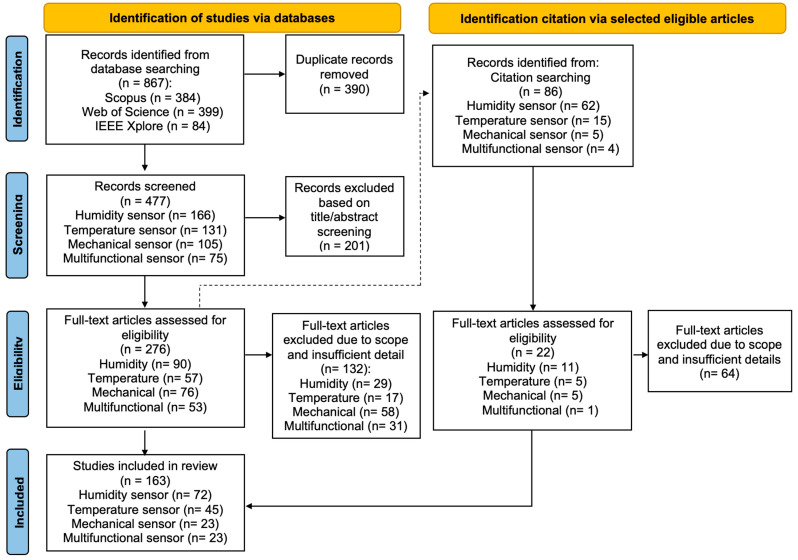
Flow diagram of the adapted PRISMA approach used in this study capturing screening step and results.

A total of 953 articles were screened, and after excluding duplicates and studies that did not meet the eligibility criteria, 163 relevant articles were identified. The complete selection process is shown in Figure 2. The PRISMA flow diagram illustrates the systematic review process, detailing the number of articles included and excluded at each stage. Among the relevant articles, 72 deal with humidity sensors, 45 with temperature sensors, 23 with mechanical sensors (10 pressure sensors and 13 strain sensors), and 23 articles related to carbon-based multifunctional sensors. Articles included in this review are largely from Journals of Quartiles, Q1 and Q2.

### 3.1. Humidity Sensors

Table 3 summarizes 72 carbon-based humidity sensors with potential application in packaging for food and medical/pharmaceutical products, detailing the materials used, production methods, and resulting properties.

Among the carbon materials studied, GO-based sensors were the most commonly used (33 studies) [45,46,47,48,49,50,51,52,53,54,55,56,57,58,59,60,61,62,63,64,65,66,67,68,69,70,71,72,73,74,75,76,77], followed by graphene (17 studies) [78,79,80,81,82,83,84,85,86,87,88,89,90,91,92,93,94], rGO (8 studies) [95,96,97,98,99,100,101,102,103], CNTs (7 studies) [68,76,104,105,106,107,108] and GQDs (4 studies) [109,110,111,112]. Sensor performance is frequently enhanced by incorporating carbon-based materials with polymers, metal oxides, specific dopants, and advanced nanostructural materials. A shift is noted from metal- to carbon-based electrodes, such as graphene [67,84,85], GO [77], rGO [97], Laser induced graphene (LIG) [53,54,62,78], carbon [113], CNTs [72], and their composites [77], to support cost reduction and improve flexibility and durability.

The fabrication techniques are diverse and tailored to different sensor configurations and specific performance. Key methods include drop-casting, spin-coating, and screen-printing created uniform films, whereas electrospinning, laser scribing, hydrothermal techniques form 3D or textured structures. The main types of investigated humidity sensors are resistive (29 studies), capacitive (18 studies), and impedance (11 studies), with a growing interest in hybrid configurations, which combine different sensing mechanisms for improved environmental adaptability.

Many carbon-based humidity sensors operate effectively across 10–97%RH, and some sensors like oxidized carbon nanohorns/graphene oxides/Tin Oxide/Poly(vinylpyrrolidone) (CNH/GO/SnO_2_/PVP) [47], GO/oxidized CNH/PVP [48], and molybdenum ditelluride/graphene (MoS_2_/graphene) [78], extended to 0–100%RH detection. In contrast, several sensors, such as N-S co-doped GQDs, have a narrow range (40–90%RH) with optimized performance [110]. Sensitivity varies significantly based on material composition and structure, ranging from 0.022 for ZnO/PVP-rGO [96] to 9,750,000% for paper cellulose fiber/GO [77].

The response and recovery time is essential for real-time monitoring and the values vary from 0.02 s for nanocrystalline graphite [114] to 333 s for TEMPO-oxidized cellulose fibers/carbon nanotubes (TOCFs/CNTs) [106]. Capacitive sensors generally show faster response times than resistive and impedance sensors. Notably, 18 sensors, including ZnO/PVP-rGO nanocomposite demonstrated faster recovery than response time [53,54,55,57,58,59,63,64,70,71,72,75,77,78,82,96,101,111].

The selectivity of carbon-based sensors is key in ensuring reliable sensor performance under real-world conditions. The reviewed studies [46,52,55,59,62,88,113] demonstrated high selectivity to water vapor through material modifications such as doping, composite integration, and surface functionalization. The reusability, inferred from stable performance under repeated humidity cycles, is indirectly supported by the stability and durability data, though direct cyclic reuse metrics have not been reported.

Sensor stability is crucial for consistent sensor performance in different transportation scenarios of products whereas low hysteresis is important for its long-term reliability. The stability was up to 1095 days for laser-reduced GO/MWCNT sensors, fabricated using a 785 nm, 5 mW laser with a 50 µm spot size under Direct Laser-Scribed (DLS) conditions [97]. The reliability remains around 3–8% for most sensors, with the exception of Li-doped GO achieving 0.83% [45]. Some sensors, such those with GO functionalized with hydroxyl groups [46], shellac-derived carbon thin film [113], oxidized CNH/GO/SnO_2_/PVP nanocomposite [47], and GO-oxidized CNH-PVP [48] consuming 15 µW [46] to 2 mW, could be ideal for prolonged monitoring. This is a very useful feature as low power consumption sensors are advantageous in many respects from a sustainability and environmental standpoint as well as for system miniaturization.

**Table 3:** Summary of different humidity sensors reported in the literature with their resulting properties including the sensing range, sensitivity, durability, linearity, response, and recovery time.

Material	Fabrication Technique	Type	Sensing Range (%RH)	Sensitivity	Response/ Recovery Time (s)	Stability (Days)	Linearity	Remarks/Comments	Ref
Li-doped GO	Drop casting	Resistive	11–97	17.13–3038.16%	4/25	Not reported (N)	Yes	Hysteresis is 0.83% and thermal stability is 850 °C.	[45]
GO functionalized with hydroxyl groups			6–95	~38.5	8.5/13	390	Yes	Hysteresis is 0.63%. High selectivity to humidity. Power consumption is 15 µW.	[46]
Oxidized CNH/GO/SnO_2_/PVP nanocomposite film			0–100	0.9021 Ω/% RH	42/164	N	Yes	CNHox/GO/SnO_2_/PVP mass ratio is 1/1/1/1. Power consumption is <2 mW.	[47]
GO-oxidized CNH-PVP			0–100	0.15–0.2	40–90/62–73	N	Yes	Optimal GO:CNH:PVP is 1:1:1. Power consumption is <2 mW.	[48]
Ultra-thin, single-layer GO film			10%–95	120.57%/%RH	0.49/0.65	60	No	Optimal sensor has 300 nm GO with 20 μm electrodes spacing.	[49]
Oxidized single-walled carbon nanohorns (SWCNHs)			10–90	~2.1 × 10^7^ Ω/RH (air)	3/N (air)	N	Yes	Surface area is 1300–1400 m^2^/g.	[115]
				~9.1 × 10^6^ Ω/RH (N_2_)	8/N (N_2_)				
GO/PVA composite		Resistive Frequency	20–80	−12,000 Ω/%RH	N	N	N	It achieves ~1.8% RH resolution.	[50]
				0.0001 kHz/%RH					
rGO/PVDF composite	Solution casting	Resistive	11–97	98.99%	21/26	90	Yes	Optimal is 30 vol% rGO/PVDF. Hysteresis is 5.5% and decomposition from 434° C.	[95]
Endohedral lithium-doped SWCNT/sodium dodecylbenzenesulfonate (Li@SWCNT/SDBS)	Arc discharge and drop casting		11–97	4%/%RH	N	N	No	Optimal sensor is five-layer thin film (~5 µm thickness). Hysteresis is 4.3%.	[104]
GQDs/Ag nanoparticles (AgNPs)	Hydrothermal and drop casting		25–95	98.14%	15/15	N	No	Optimal GQDs/AgNPs is 1:1.	[109]
GO film	Drop casting	Capacitive	15–95	37,800%	10.5/41	30	Yes	Hysteresis is ~5%.	[51]
GO/Ag composite			11–97	25,809 pF/%RH	~8/~12	30	N	Optimal Ag content is 2 wt%. Good selectivity for H_2_O vapor.	[52]
GO			0–97	1800 pF/% RH	16/9	N	Yes	Spiral LIG as electrodes. Optimal GO thickness is 50 nm. 3.03% hysteresis.	[53]
GO			10–90	3862 pF/%RH	58/15	42	N	Hysteresis is 1.2%. Optimal sensor used 60 µL GO and 150 µm gap size for LIG interdigitated electrodes (IDE).	[54]
GO/MoTe_2_ composite nanosheets			11.3–97.3	94.12 pF/%RH	39/12	35	N	Optimal GO to MoTe_2_ ratio is 1:2. High humidity selectivity.	[55]
ZnO/PVP-rGO nanocomposite			15–95	~0.022	~12/~3	87	Yes		[96]
GO-Mn-doped ZnO nanocomposite		Capacitive	10–90	N	4.5/21	30	Yes	95.7 times higher sensitivity in capacitance and 97 times in resistance compared to conventional GO.	[56]
		Resistive							
GO-doped P(VDF-TrFE)/LiCl composite		Capacitive change	25–95	1708.8 pF/%RH	7.8/4.5	N	Yes	Pores from 300 nm to 1.1 µm. Reduced hysteresis due to GO and LiCl modification.	[57]
GO		Resonant frequency	10–90	0.719 kHz/%RH	<78/54	30	No	Resolution (0.4% RH), hysteresis (<4%), and minimal response to CO_2_.	[58]
HGO/GO/Mg^2+^ composite membrane			11–97	0.0343 kHz/%RH	7/6	10	Yes	Hysteresis is ~3.2% RH. High humidity selectivity.	[59]
GO		Voltage	33–98	1.1–10.0 mV/%RH	0.28/0.3	2.5	Yes	GO thickness is 10 μm.	[61]
2D MoS_2_/graphene nanocomposite foam		Impedance	0–100	50,000– 385,000 Ω/%RH	4/2	N	No	Sensor used LIG as electrodes. Hysteresis is 3.8%.	[78]
Laser-reduced GO/MWCNT	Drop casting and direct laser scribing	Impedance	11–97	350,000 Ω/%RH	0.061/2.3	1095	Yes	Sensor used rGO IDE. Hysteresis is 3.1%.	[97]
		Capacitance		798 pF/%RHc					
Thermally reduced GO	Spin-coating	Resistive	32–65	5%	35/N	N	Yes	Highly thermal-reduced GO has the optimal performance.	[98]
P(VDF-TrFE) with graphene flower composite		Capacitance Impedance	8–98	0.0558 pF/% RH	0.8/2.5	15	Yes	N	[79]
GO		Impedance	6–97	182,068.791/%RH	0.8/0.9	1	Yes	Ti_3_C_2_T_x_ MXene-based sensor exhibited faster response than sensors using metallic electrodes.	[60]
N-S co-doped GQDs	Hydrothermal and spin-coating		40–90	N	15/55	90	N	Optimal GQDs content is 10 mg with 2.2% hysteresis.	[110]
GQDs/carbon nitride (g-C3N4) composite			7–97	100,000 Ω/RH	44 /10	N	Yes	Low hysteresis (<1%) and high surface area (545 m^2^/g)	[111]
Bi-layered PVA/graphene flower composite film	Spin-coating and spray-coating	Capacitance	40–90	29,000 pF/%RH	2/3.5	15	N	Uniform dispersion of PVA/GF layer with ~2.32 µm thickness.	[80]
		Impedance							
Shellac-derived carbon (SDC) thin film	Spray coating and thermal annealing	Resistive	0–90	0.54/% RH	0.14/1.7	28	Yes	Carbon IDE. High selective to humidity. Power consumption is ~1 mW.	[113]
rGO-sodium dodecyl sulfate (SDS) composite film	Drop-coating	Resistive	25–95	11.4143 Ω/% RH (RT)	9/10	10	Yes	Hysteresis is 0.04852%.	[99]
GO			11–97	1.113 Ω/Ω–%RH	2/35	N	Yes	Sensor used 300 nm wrinkled GO film on the LIG electrode. Hysteresis is 3%. High humidity selectivity.	[62]
GO		Quartz crystal microbalance (QCM)	11.3–97.3	0.1605 kHz/%RH	30/5	N	Yes	The study used the finite element analysis software COMSOL Multiphysics.	[63]
Polydopamine-coated cellulose nanocrystals/GO nanocomposite (PDA@CNC/GO)	Drop-coating	Resonance frequency	11.3–97.3	0.05466 kHz/% RH	37/5	21	N	Optimal composition is 30 wt% PDA@CNC. Hysteresis is 4.3% RH.	[64]
Graphene flower/ZnO composite	Sol–gel and spray-coating	Resistive	15–86	7.7 µA/%RH	0.4/4	N	N	High surface area to volume ratio and pore composite.	[81]
GO on tilted fiber grating (TFG)	Dip-coating	Resonance wavelength Intensity	30–80	0.0185 nm/%RH	0.042/0.115	N	Yes	GO thickness is 54 nm.	[65]
GO/PVA composite film		Intensity	20–99.9	0.529 RH (%)	147/293	N	Yes	N	[66]
Graphene–carbon ink	Screen printing	Resistive	25–91.7	12.4 Ω/%RH	~31/~8	120	N	Optimal configuration is single-layer sensor.	[82]
G/polypyrrole/carbon black (CB) composite			23–92.7	12.2 Ω/%RH	5/7	21	N	Durability is 100 bending cycles Single-layer is the most effective configuration.	[83]
Graphite/WO_3_ nanocomposite			11–97	12.7–60.8%	N	N	Yes	Optimal sensor using graphite/WO_3_ ratio is 1:3, with <1% hysteresis. 120° bending angles.	[116]
Multilayer GO		Resonance frequency Backscattered phase	11–98	0.5°/%RH	N	N	N	30 µm GO film and printed graphene antenna electrodes.	[67]
Cellulose nanofiber (CNF) and graphene nanoplatelet (GNP) composite	Mixing and screen printing	Resistive	30–90	240%	17/22	240	N	Composite with 200 mg GNP as electrode.	[84]
Graphene ink	Inkjet printing	Capacitive	10–70	0.03 pF/%RH	2.46/2.63	10	N	Optimal sensor is six-layer graphene film with graphene IDEs.	[85]
GO/CNT−OH/Nafion nanocomposite		Resonance frequency	30–95	547 kHz/%RH	110/115	2.08	Yes	Hysteresis is 3%.	[68]
Functionalized MWCNTs and hydroxyethyl cellulose (HEC) composite	Gravure printing	Resistive	20–80	0.0485/%RH	20/35	0.4	Yes	The optimal FMWCNT concentration is 2.5 wt%.	[105]
Carboxymethyl cellulose@graphene (CMC/G) composite	3D printed groove mold	Impedance	11–95	97%	300/N	16	Yes	Optimal graphene content is 0.16 wt%.	[86]
Graphene film	Liquid phase exfoliation and LB assembly	Resistive	8–95	5%	0.028/0.03	N	Yes	The thickness is ~3.4 nm (~ 10 layers). Flexibility is 10° bending.	[87]
GO	Self-assembly	Capacitive	30–90	0.00565 pF/% RH	180/N	14	N	Optimal sensor is 2 mg/mL GO with 2.85% hysteresis.	[69]
Pyranine modified-rGO composite	One-step supramolecular assembly	Impedance	11–95	IL/IH = 6000	<2/~6	N	Yes	Hysteresis is 8% RH. Stable for 100 cycles.	[100]
TEMPO-oxidized cellulose fibers (TOCFs)/CNTs	Electrostatic self-assembly	Current	11–95	87%	333/523	90	Yes	Optimal TOCFs-to-CNTs ratio is 30:1 with a thickness of 48.2 µm and 7.3% hysteresis.	[106]
G with 3D flower-like ZnO composite	Hydrothermal	Impedance	12–90	446	120/160	30	N	Optimal G content is 70 wt% with 2.32% hysteresis. High humidity selectivity.	[88]
PVDF (polyvinylidene fluoride) with 0.5 wt% G	Electrospinning	Capacitive	35–90	0.0463 pF/%RH	N	N	Yes	PVDF/G with Ag electrode, showed 21.3 times faster than DHT11.	[89]
SnO_2_/rGO nanocomposite			11–95	37,491%	80/4	N	N	Optimal rGO doping content is 2 wt%. Durability is 1000 bending cycles.	[101]
BP/G hybrid	Electrospray	Resistive	15–70	43.40%	9/30	28	Yes		[90]
GO	Electrospray deposition	Resonant frequency	11–97	1.74%/%RH	54–68/12–22	30	No	Low thermal noise. Optimal is 250 MHz sensor.	[70]
Holey-reduced graphene oxide (HRGO)	H_2_O_2_-etching-reaction-assisted hydrothermal	Impedance	11–97	−0.04317 log Z/%RH	<3/29	28	Yes	Surface area is 274.5 m^2^/g. Hysteresis is 2.57%.	[102]
GO	Dripping and vacuum heating	Capacitive	20–90	1.77–164.98 pF/% RH	10/2	N	N	Hysteresis is 1%.	[71]
GO	Dripping and coating		10–90	16.7 pF/%RH	0.0208/0.0199	80	N	The optimal sensor used 1 mg/mL GO and CNTs as electrodes. Hysteresis (<0.44%).	[72]
ZnO nanowires and GQDs composite	Dripping	Resonance frequency	30–90	40.16 kHz/%RH	~30/~35	N	No	Optimal GQDs content is 2 mg/mL. 30° bending angle.	[112]
SWCNTs	Vacuum filtration	Resistive	15–98	246.90%	290/510	N	Yes	Optimal sensor is suspended aligned. SWCNT beams, with 36 μm suspension lengths.	[107]
rGO/PANI composite	Filtration		0–98	580%	~70/~139	N	Yes	Hysteresis is 3%. Optimal rGO to PANI ratio is 5%.	[103]
Laser-induced graphene (LIG)	Laser Direct Writing (LDW)	Capacitive	30–90	N	8/10	N	No	The porous, hair-like LIG pattern was designed with 2-CAD.	[91]
Light-scribed GO	Laser scribe	Impedance	7–97	1.67 × 10^6^ Ω/%RH	N	1	Yes	Hysteresis is 0.3–7%.	[73]
G/ZrO_2_ nanocomposite	Sol–gel		12–90	4011	5/20	6	Yes	Hysteresis is <1.95%. Optimal is 40 wt% G/ZrO_2_	[92]
3D graphene foam	Modified Hummers’ method	Resistive	0–85.9	N	0.089/0.189	N	N	Energy structure of 3DGF model analyzed via CASTEP in Materials Studio 8.0.	[93]
Nanocrystalline graphite	Plasma-enhanced CVD	Resistive	15–85	0.0334%/%RH	0.02/N	N	Yes	Hysteresis is 5%. It is meandered strip structure.	[114]
SWCNT	Immersion		20–80	54.7% (s-CNT)	40/100	N	Yes	Hysteresis is 11.45% (semiconducting-CNT) and 0.31% (metallic-CNT).	[108]
				2.9% (m-CNT)					
G/p-aminophenol/poly-2-hydroxyethyl acrylate (G/p-AP/PHEA)	In situ free-radical polymerization		0–94	29%	N	N	N	N	[94]
Etched GO film	Etching	Capacitive	10–100	0.000106 pF/% RH	1.011/N	N	N	The study using COMSOL Multiphysics.	[74]
Nanofibrillated cellulose (NFC)/GO/PDMS aerogel composite	Ultrasonic dispersion and freeze-drying		11–97	6576.41 pF/% RH	57/2	N	No	Porosity is 99.6%.	[75]
GO/MWCNTs hybrid on tilted Fiber Bragg Grating (TFBG)	Physical precipitation	Optical fiber Amplitude	30–90	0.377 dB/%RH	4/N	N	Yes	Hysteresis is 0.7%.	[76]
Paper cellulose fiber/GO matrix (PCFGOM)	N	Impedance	10–90	9,750,000% (1 kHz)	1.3 /0.8	1	Yes	The sensor used 0.15 w/w% PCFGOM as active layer and 20 w/w% PCFGOM as electrode layers.	[77]
		Capacitance		1,442,500% (10 kHz)					

### 3.2. Temperature Sensors

Table 4 summarizes 45 carbon-based temperature sensors with their composition, production methods, and resulting properties. Graphene-based sensors were predominant among the reported studied (19 studies) [117,118,119,120,121,122,123,124,125,126,127,128,129,130,131,132,133,134,135], followed by rGO (11 studies) [123,136,137,138,139,140,141,142,143,144,145], CNTs (9 studies) [146,147,148,149,150,151,152,153,154], and GO (4 studies) [155,156,157,158]. Other carbon materials, including GQDs [159], carbon dots (CDs) [160], and amorphous carbon [161] were reported in only one study, respectively, highlighting their limited exploration in this field. The incorporation of polymers and metals/metal oxides into carbon-based materials has been extensively explored to enhance the performance of temperature sensors. The combination with metal/metal oxides were predominantly applied to rGO [138,141,145] and graphene [128]. In contrast, polymers like Poly(3,4-ethylenedioxythiophene) polystyrene sulfonate (PEDOT:PSS), polydimethylsiloxane (PDMS), and gelatine were widely combined with various carbon materials, including graphene (6 studies) [118,119,120,121,122,135], GO (2 studies) [157,158], rGO (3 studies) [140,143,144], and CNTs (5 studies) [146,147,148,150,151].

Chemical vapor deposition (CVD) was the most dominant method (10 studies) [123,127,128,129,130,131,132,151,152,153], particularly for graphene and CNT sensors. Methods of coating (12 studies) [117,118,119,120,121,122,123,124,136,137,146,155] including spray coating [117,123,124,136], drop casting (5 studies) [138,147,148,155,156], and printing (6 studies) [139,140,141,149,150,157] was widely used in rGO, GO, and composite sensors, offering simplicity and scalability.

Graphene-based sensors fabricated via CVD exhibited the broadest detection ranges, spanning from −266.55 °C [129] to 302 °C [130], quick response time of ~0.030 s in multilayer graphene [129], and high sensitivities, such as 2.15 Ω/°C in micro-fabricated single-layer graphene [127]. Similarly, rGO-based sensors demonstrated wide detection range of −196.15–299.85 °C in rGO sensor [137], with exceptional sensitivity values of up to 1999%/°C for GQDs/rGO/alumina composite [142]. In contrast, GO-based sensors [155,156,157,158] and CNT-based sensors [146,147,148,150,151,152,153] generally operate within narrower ranges, typically starting at 20 °C, limiting their suitability for applications in chill- and cold-chain environments. However, functionalization and advanced fabrication methods have shown potential in improving detection capabilities. For instance, carboxyl-SWCNTs achieved a range of 0–80 °C but showed slow response (176.4 s) and recovery time (316.8 s) [154] whereas CNTs produced using gravure printing demonstrated a broader temperature range (−40–100 °C) and fast response (0.3 s) and recovery time (4 s) [149].

Response times varied significantly from 0.030 s for multilayer graphene produced by CVD [129] to 306 s for uncovered drop-casted GO sensors [155] depending on the material and encapsulation approach. Composite systems often outperformed pure carbon materials in detection range, sensitives, and responsiveness, particularly those combined with metal or metal oxides. For example, rGO/Ag exhibited extended temperature sensing ranges (−60–80 °C), faster response times (0.47 s), and good sensitivity (0.555 Ω/°C) [138].

Long-term stability was assessed in 10 studies [121,126,138,140,143,146,147,152,156,158], rGO/Ag nanocomposite with Parylene encapsulation demonstrating exceptional longevity, maintaining performance over 730 days durability [138].

**Table 4:** Summary of different temperature sensors reported in the literature with their resulting properties including sensing range, sensitivity, stability, response, and recovery time.

Material	Fabrication Technique	Sensing Range (◦C)	Sensitivity/TCR (%/°C)	Response/ Recovery Time (s)	Stability (Days)	Remarks/Comments	Ref
GO	Drop casting	20–70	822 Ω/°C	306/554 (uncovered)	N	Encapsulation: PDMS	[155]
	Spray coating	20–60	N	0.525/0.35 (uncovered)			
				5.18/9.68 (covered)			
rGO	Spray coating	30–100	0.6345%/°C	1.2/N	N	Encapsulation: high-temperature transparent insulating tape.	[136]
Multilayer graphene ink film		30–90	43.27 μV/K	0.15 /15	N	Optimal sensors have 108 nm thickness and provide 300 μV output voltage, and signal-to-noise ratio is 35.	[117]
rGO	Spin coating	−196.15–299.85	−0.801–−32.04%/°C	52/285	N	Optimal rGO concentration is 3wt%, with 0.1 °C resolution.	[137]
MWCNT doped in polyethylene glycol and PU (MWCNT-PEG-PU) nanocomposites		25–50	~80%	N	7	Optimal MWCNT concentration is 8 wt% and stable 30 bending cycles.	[146]
Graphene-coated microfiber (GCM)	Coating	22–40	2.1 dB/°C	N	N	Minimum resolution is 0.0005 °C.	[118]
Polyaniline/graphene (GPANI) embedded in Polyvinyl Butyral (PVB) composite film	Coating using Mayer rod	25–80 °C	−1.2%/°C	N	N	Sensor also responds to external pressures (0–30 kPa). Encapsulation: Bezel tape	[119]
Graphene and gelatin nanocomposite	Blade coating	−13–37	−5.3–−23 mV/°C	10.4/N	N	Stable for 20 cycles.	[120]
Graphene/gelatin nanocomposite		−13–37	−19 mV/K	41.8/N (pristine sensor)	2	Energy consumption is 8.1 μWh for pristine sensor.	[121]
				28.9/N (aged sensor)		Energy consumption is 8.5 μWh for aged devices.	
PU/G Nanocomposite	In situ polymerization and dip coating	25–60	6 pm/°C	N	N	Thermal stability to 217 °C from 204 °C.	[122]
rGO	Air brush spray coating	0–100	45.1%	121/N	N	N	[123]
Graphene nanoplatelets (GNP)			52%	89/N			
Plasma-grown graphene (Gpl)	Plasma discharge		20.5%	125/N			
Graphene via CVD (Gcvd)	CVD		27%	68/N			
GO	Post-COMS MEMS Drop casting	−70–40	155.73–58,555.26 pF/°C	Not reported (N)	30	Capacitance sensor.	[156]
rGO/Ag nanocomposite	Ultrasonication and drop casting	−60–80	0.555 Ω/°C	0.47/N (cold)	730	Encapsulation: Parylene.	[138]
				3.45/N (hot)			
CNT/PEDOT:PSS composite	Drop casting	25–45	−1.97%/°C (initial)	N	6	Encapsulation: PDMS Optimal CNT/PEDOT ratios is 1:5.	[147]
			−2.80%/°C (6 days aging)				
CNT and methylcellulose (CNT/MC) composite	Solution casting	20–70	0.2%/°C	6.1/3.1 (hot) 5.2/7.2 (ice)	N	Stable over 480 cycles.	[148]
Graphene Nanoribbons (GNRs)	Mask spraying or direct handwriting	30–80	172% TCR = 1.27%/°C	0.5/0.5	N	Using MWCNT ink electrodes and Scotch tape encapsulation. 0.2 °C resolution and stable 5000 bending cycles.	[124]
CNT	Gravure printing	−40–100	−0.4%/°C	0.3/4	N	High accuracy (±0.5 °C). Encapsulation: organic and silver.	[149]
GO/PEDOT: PSS composite	Mask printing	25–100	−1.09%/°C	18/32	N	Encapsulation: Kapton tape. Stable 1000 bending cycles.	[157]
Functionalized and reduced graphene oxide via sulfonated aromatic diamine (f-rGO)	Inkjet printing	30–82	−0.0164/°C	176.4/316.8	N		[139]
CNT/PEDOT-PSS composite		25–50	0.31%/°C	~39/~196	N	Encapsulation: translucent polyurethane welding tape. Stable 1000 cycles bending.	[150]
rGO with alkali lignin		25–135	0.59%/°C	N	180	Sensor used meander-shaped rGO as electrode.	[140]
rGO/Ag	Aerosol jet printing	0–200	0.001162–0.001519/°C	N	N	Optimal four layers rGO/Ag. Stable 1000 bending cycles.	[141]
Porous LIG	CO_2_ laser-induced Direct laser writing	1–8	N	16/58	N	Encapsulation: PDMS. Stable 200 bending cycles.	[125]
LIG	Laser direct writing	24–80	−0.58%/°C	N	14	Sensors optimized by finite element analysis photothermal model.	[126]
Amorphous carbon films	DC Magnetron Sputtering	20–150	1.62 mV/°C TCR = 0.00128/°C	N	N	DC magnetron sputtered sensors are more stable and practical than ion-beam-deposited sensor.	[161]
Micro-fabricated single-layer graphene	CVD	10–30	1.25 Ω/°C (SiO_2_/Si substrate)	N	N	Sensor used graphene electrodes and PDMS gasket encapsulation.	[127]
			2.15 Ω/°C (SiN substrate)				
			1.90 Ω/°C (suspended graphene substrate)				
CNT forest-PDMS composite		30–90	0.55 Ω/°C	N	N	Encapsulation: PDMS.	[151]
Graphene and Lithium Niobate (LiNbO_3_)		10–70	−0.23 nm/°C	N	N	Encapsulation: PDMS.	[128]
Multilayer graphene		−266.55–26.85	−1 (THS < −243.15 °C)	~0.030/N	N	Sensor made by seven layers of single-layer graphene.	[129]
			<0.1 (THS > −173.15 °C)				
Vertically aligned CNT film	TCVD	20–110	4.74 μA/°C (air)	N	30	Triple-electrode structure enables long-term sensor operation.	[152]
			22.72 μA/°C (N_2_)				
MWCNT	CVD and wet transfer	22–200	0.0033 V/°C TCR = 0.00103/°C	N	N	2.7 μm MWCNT sensor had carrier mobility (−28.5574 cm^2^/Vs).	[153]
Single-layer graphene		27–302	0.00207/°C (27–177 °C)	N	N	Resistance is almost unaffected by humidity.	[130]
			0.00239/°C (177–302 °C)				
Suspended few-layer and multilayer graphene		25–120	1.07–3.5%/°C	N	N	N	[131]
Graphene	CVD and AI sacrificial layer process	25–200	2.134 Ω/◦C	N	N	Enhanced 41.93% consistency. Encapsulation: SiO_2_ layer.	[132]
GQDs embedded in a rGO/alumina composite film	Sol–gel	−196.15–26.85 26.85–99.85	−1999%/°C −0.98%/°C	~0.3/0.8 3.96/6.01	N	Short-term stability is 50 cycles.	[142]
CNC-assisted carbon dots (CDs)-grafted SrAl_2_O_4_: Eu^2+^, Dy^3+^ (SAO) phosphors composite film	Sol–gel and vacuum filtration	−30–110	0.257	N	N	Short-term stability is 3.5 cycles.	[160]
High-strength metallurgical graphene (HSMG)	Modified PMMA-based transfer	−253.15–21.85	−0.007/°C	N	N	Encapsulation: transparent polymer.	[133]
Polyethyleneimine/reduced graphene oxide (PEI/rGO)	Spray dipping	25–45 0–60	1.3%/°C	0.33–0.443/N	120	Encapsulation: PDMS. 0.1 °C resolution and 500 bending cycle stability.	[143]
GO/PEDOT: PSS micro/nanowires	Soft lithography	30–80	−0.007599/°C	3.5 /13.4	30	Optimal GO doping ratio is 13:1.	[158]
LIG	CO_2_ laser irradiation	30–60	−0.04145%/°C	30/N	N	High measurement accuracy (±0.15 °C).	[134]
PDA-rGO/sodium alginate/polyacrylamide composite organohydrogel	Solvent displacement and cross-linking	−20–60	97.6%/°C (−20–−5 °C)	0.2/0.3	N	Encapsulation: VHB tape. Stable over 3 h.	[144]
			10.57%/°C (−5–15 °C)				
			1.45%/°C (15–60 °C)				
Star-like rGO/SnO_2_/Co_3_O_4_ composite	Facile wet chemical precipitation	25–125	0.561%/°C	N	N		[145]
GNP/PDMS nanocomposite	Three-roll milling and molding	30–80	0.052–11.7/°C	N	N	The optimal GNP concentration is 6 wt%.	[135]
Carboxyl-SWCNTs	N	0–80	−225 Ω/°C	N	N	Encapsulation: thermos-reversible polymer. Self-healing 30 bending cycles.	[154]
GQDs/hollow-core fiber	N	10–80	−0.01375/°C	N	N	N	[159]

### 3.3. Mechanical Sensors

The review revealed 10 carbon-based pressure sensors that could be used to monitor internal and external forces exerted on packaging as well as 13 carbon-based strain sensors. These findings are summarized in Table 5 and Table 6, respectively.

Similarly to temperature sensors, graphene-based sensors dominate the literature (4 studies) [162,163,164,165] while other carbon materials, including GO [166], rGO [167], CNTs [168], carbon-ink [169], and CB [170] were reported in only one study each. A total of 70% of sensors were porous composite materials that mainly harness the high conductivity and mechanical strength of carbon materials alongside the flexibility, stretchability, and durability of polymers. However, some pressure sensors were produced with only graphene films via CVD [162,163]. The sensor production methods range from simple techniques such as molding [164,167,171], dip coating [169], self-assembly [166], and solvent extraction [165] to relatively more advanced fabrication techniques such as laser thermoforming [170], electrospinning and mechanical drawing [168].

The sensing range depends on materials and structure design, spanning from 20 kPa [167] to 20,000 kPa in a graphene N/MEMS mechanical sensor with crossbeam structure [163]. Porous structures such as porous PDMS [171], graphene/PDMS sponge [164], and polyurethane/graphene (PU/G) foams [165] reach up to 500 kPa, while softer materials such as tannic acid-reduced graphene oxide combined with polyvinyl alcohol (TA-rGO/PVA) hydrogel [167] and nitrogen-doped graphene oxide/dopamine/polyaniline (GO/DA/PANI) aerogel [166] are limited to 20–25.48 kPa, which restricts their use to low-pressure environments and modified atmosphere packaging. Film structures like monolayer graphene have narrow sensing range, up to 80 kPa [162].

The sensor sensitivity also varies, with the formulation and production conditions ranging from 0.0259 kPa^−1^ [169] to 2200 kHz/kPa [164]. High sensitivity was observed at low pressures and this decreased as the pressure increased, particularly in composites with wide detecting range. Indeed, the sensitivity of a graphene/PDMS composite decreases from 2200 kHz/kPa at 0–10 kPa to 37.5 kHz/kPa at 200–500 kPa [164]. The incorporation of carbon black and use of glucose monohydrate to form porous PDMS/graphene composite contributed to improved flexibility and sensitivity (109.4 kPa^−1^) of the sensor [170]. Fluoropolymers such as Poly (vinylidene fluoride) (PVDF) and its copolymer poly(vinylidene fluoride-trifluoroethylene) (P(VDF-TrFE)) with SWCNTs or MWCNTs have also been used with reasonable sensitivity. However, recycling or reusing PVDF components when they reach the end of their useful lives is particularly challenging, whereas their disposal by incineration poses an environmental issue because of the potential formation of hydrogen fluoride at elevated temperatures [172].

The durability varies from 100 cycles for hydrogels [167] to 10,000 cycles for porous composites like PDMS/graphene [171]. Response and recovery times varied across materials and sensor structures, and graphene/PDMS sponge achieved fastest response time of 7 ms with 60 ms recovery time [164], while porous PDMS with MWCNT/PEDOT electrode showed 1 s of response and recovery time [171].

**Table 5:** Summary of different pressure sensors reported in the literature with their fabrication methods and resulting properties including the sensing range, sensitivity, durability, response and recovery time.

Material	Fabrication Technique	Sensing Range (kPa)	Sensitivity/Gauge Factor (GF)	Response/ Recovery Time (s)	Durability (Cycles)	Remarks/Comments	Ref
Suspended monolayer graphene (G)	CVD	0–80	GF = 6.73 (circular membrane)	Not reported (N)	Not reported (N)	An improved theoretical model was developed to predict GF and confirm their independence of doping concentration and graphene crystallographic orientation.	[162]
			GF = 3.91 (rectangular membrane)				
Graphene	Plasma-enhanced CVD	0–20,000	0.03313 mV/V/kPa	N	35 days	Encapsulation: Si_3_N_4_ film. Error of hysteresis (2.0119%), nonlinear (3.3622%), and repeatability (4.0271%).	[163]
			GF = ~1.35				
Graphene/PDMS sponge	Mixing and molding	0.005–500	37.5–2200 kHz/kPa	~0.007/0.06	5000	LC technology used for long-distance wireless transmission. Optimal graphene concentration is 20%.	[164]
Porous PDMS	Sugar-cube mold	0–1200	360–1120 kPa^−1^	1/<1	10,000	Sensor used MWCNT/PEDOT composite electrode and low-pass filter.	[171]
Tannic acid (TA)-rGO/PVA hydrogel	Sonication, molding via freeze–thaw	0–20	2.2695 kPa^−1^	0.67/0.84	100	Optimal concentration is 2 mg/mL and tensile strength is 440.213 kPa.	[167]
Carbon ink-coated filter paper	Dip coating	0.1–100	0.0259–0.627 kPa^−1^	N	4000	N	[169]
PU/G foams	Solvent extraction	0–500	0.05–7.62 kPa^−1^	0.81/0.81	1000	Optimal graphene content is 30 wt%.	[165]
Nitrogen-doped GO, dopamine, and polyaniline composite aerogel	Self-assembly, freeze-drying, and thermal annealing	0–25.48	0.10 kPa^−1^	N	150	The optimal mass ratio of GO:DA:PANI is 5:2:2, with 1.46% nitrogen.	[166]
P(VDF-TrFE) matrix with MWCNTs	Electrospinning and mechanical drawing	5–50	~540 mV/N	N	N	Self-powered sensor achieved piezoelectric coefficient of 50 pm/V with 98% linearity.	[168]
PDMS/CB/graphene nanosheets	Laser thermoforming	0–100	109.4 kPa^−1^	0.079/0.055	5000	CB as an endothermic agent and glucose as a porogen.	[170]

Table 6 summarizes 13 carbon-based strain sensors with potential application in food and medical packaging-based monitoring during transportation.

Among the 13 carbon-based strain sensors with potential for application in food and medical packaging-based monitoring during transportation/distribution, CNTs (seven studies) [173,174,175,176,177,178,179], rGO (four studies) [167,177,179,180], and graphene (three studies) [181,182,183] are widely used. These materials were often combined with different highly stretchable and durable polymers or elastomers (e.g., PDMS, PVA, PEI, chitosan, and agar) to improve flexibility and robustness. Several sensors were made of functionalized materials, such as carboxyl-functionalized CNTs [176], polyetherimide-rGO [180], and tannic acid-modified rGO [167], which provided unique features such as self-healing and biocompatibility. Carbon-based strain sensors were generally in the form of layered and 3D composite structures employing hybrid and functionalized materials to enhance sensor performance. Multilayer designs, such as few-layer graphene films [181] has shown to improve sensor sensitivity and mechanical stability. Three-dimensional composites including fragmentized rGO sponge [177] and rGO/MWCNT composites [179] broaden the detection range while also enhancing sensors sensitivity.

Simple and relative cheap methods such as single-step Marangoni self-assembly [182], layer-by-layer assembly [180], sonication [183], and solution casting [175] were used to produce strain sensors which demonstrated low-strain detection (up to 10%) with high sensitivity. Advanced techniques, including microelectromechanical system-assisted electrophoretic deposition (EPD) [173], embedded 3D printing [184], direct writing [179], and screen printing [181] enabled the production of sensors with precise control and broader detection range.

Just for other sensor types, the strain-sensing range varies significantly with material and sensor architecture. The values spanned from 2% in the ultrathin graphene film sensor [182] to 1000% in the carboxyl-functionalized CNTs sensor [176]. Polymer-free CNT sensors fabricated via CVD demonstrated a sensing range of 0–42,100 kPa with high gauge factor (1461), suitable for high-strain applications [174]. Composite sensors’ detection range depends on polymers/elastomers flexibility or stretchability as well as the filler concentration. This ranged from 2% for chitosan/graphene [183] to 280% for TA-rGO/PVA [167]. Functionalized materials, such as carboxyl-functionalized CNTs provided high stretchability and self-healing capability, achieving up to 1000% [176]. Layered and network structure CNT/PDMS sensors achieved up to 100% strain [178].

The durability of carbon-based strain sensors varies significantly, from 100 [167] to 10,000 cycles [175]. The CNT/Agar composite sensor was particularly durable, withstanding up to 10,000 cycles, and demonstrated an enhanced strain range and sensitivity due to increased filler concentrations [175]. The TA-rGO/PVA hydrogel strain sensor exhibited quick response and recovery times (670 ms and 840 ms, respectively), but its soft nature limits its durability to 100 cycles [167].

Only four articles [167,175,177,178] reported any response and recovery time and they vary from 20 ms for FGS/AgNPs/SBS composite [177] to 670 ms for TA-rGO/PVA hydrogel sensor [167].

**Table 6:** Summary of different strain sensors reported in the literature with their fabrication methods and resulting properties including the sensing range, sensitivity, durability, response, and recovery time.

Material	Fabrication Technique	Sensing Range (%)	Sensitivity	Response/ Recovery Time (ms)	Durability (Cycles)	Remarks/Comments	Ref
Patterned MWCNT/PDMS	Microelectromechanical system-assisted EPD	0–14	13–120	N	N	Sensitivity tailored by MWCNT film thickness and entanglement. Sensor adapted to an arbitrarily curve surface.	[173]
Polymer-free CNTs	Hot-wall atmospheric CVD	0–42.1 MPa	1461	N	N	Higher sensitivity in IDE devices than single-gap electrodes.	[174]
CNT/Agar composite	Solution casting	0–118	0.28	160/250	10,000	Increasing filler concentration improved strain from 0.8 to 1.1, and stress from 35.2 to 45.8 kPa.	[175]
PDMS-TDI (2,4′-Tolylene diisocyanate)-carboxyl-functionalized MWCNTs nanocomposite	One-pot synthesis, ultrasonication, and casting	0–1000	0.65–2.43	N	1000	Sensor had 98.1% self-healing efficiency at 60 °C over 9 h.	[176]
Fragmentized rGO sponge (FGS)/AgNPs/polystyrene-butadiene-styrene (SBS) composite	Multiple-step process	0–120	20.5–1.25 × 10^7^	20/N	2000	Microcrack contributed to sensitivity. Sensor had 1521 S/cm conductivity and 680% break elongation.	[177]
CNTs/PDMS		0.007–100	87	65/N	1500	Optimal sensor had network cracks and 15 layers of CNT.	[178]
rGO/MWCNTs composite	Direct writing printing	10–40	18.55	N	900	N	[179]
Carbon grease	Embedded 3D printing	400	3.8	N	1000	Up to 10% variation from its original value after large strains.	[184]
Few-layer graphene	Mechanical exfoliation and screen printing	0–6	20.02	N	100,000 flexing cycles	Number of prepared graphene layers was 2–5 layers.	[181]
					1000 abrasion cycles		
TA-rGO/PVA hydrogel	Sonication and molding via freeze–thaw cycles	0–280	1.936 78	670/840	100	Optimal concentration is 2 mg/mL and tensile strength is 440.213 kPa.	[167]
PEI-rGO nanocomposite	LBL self-assembly	0–5 (~800 kPa)	N	N	500	Rapid self-healing (~10 s), and 98% efficiency at room temperature.	[180]
Ultrathin graphene film	Single-step Marangoni self-assembly	2	1037	N	N	Optimal thickness is 4.4 nm with 3.4% failure strain.	[182]
Chitosan-graphene	Bath sonication and vacuum filtration	0–2	18.6	N	N	Chitosan-G had better graphene electrical properties than pullulan and alginate.	[183]

### 3.4. Multifunctional Sensor

Table 7 summarizes 23 carbon-based multifunctional sensors with potential applications in packaging food and medical or pharmaceutical products. Among the studies reviewed, 9 articles examined dual-functional sensors, detecting two stimuli simultaneously [185,186,187,188,189,190,191,192,193], 11 explored triple-functional sensors [194,195,196,197,198,199,200,201,202,203,204], and 3 quad-functional sensors, focusing on humidity, temperature, pressure, and strain stimuli [205,206,207]. Graphene, rGO, and CNT-based material were predominantly used, often in combination with different polymers or other materials such as carbon black to enhance the multifunctionality of the sensors. Fabrication methods often involve coating, CVD, molding, and printing, allowing for simple and scalable production. Many sensors incorporated multiple fabrication techniques to optimize performance.

The selective reactivity to specific stimuli was achieved by using different carbon materials or compositions [187,188,190,191,194,203]. For example, Bae et al. (2018) fabricated a dual-mode sensor that utilized SWCNTs/PDMS for pressure sensing (0–25 kPa) and rGO for temperature sensing (22–70 °C) with good sensitivity (0.7/kPa and 0.83%/°C, respectively), quick response (0.05 s and 0.1, respectively), and stability over 10,000 cycles [187]. Similarly, all carbon-based sensors with carbon nanocoils (CNCs) and CNTs for simultaneously sensing temperature, humidity, and strains have been developed by Li et al. [203]. The authors achieved a wide detection range from −266.15 to 126.85 °C (temperature), 10% to 80% (relative humidity) and up to 100% strain with high strain resolution (0.01%) and fast response time (16 ms) alongside a stability of 10,000 cycles. Li et al. [192] developed a dual-mode temperature and strain sensor based on graphene/PEDOT:PSS hydrogel, with a detection range of 7–60 °C for temperature and up to 1000% for strain. The sensor demonstrated high sensitivity (gauge factor 8.1 for strain, −7.16%/°C for temperature), fast response (0.2 s), and stability over 10,000 cycles.

Other reported multiple sensing systems showed a temperature range starting from 20 °C [187,188,189,190,193,197,198,202,204,205,206] or lower pressure range below 0.6 kPa [195,201], limiting their application for packaging when considering the stresses encountered in the transportation/distribution chain.

**Table 7:** Summary of different multifunctional sensors reported in the literature with their modes, fabrication methods, and resulting properties including the sensing range, sensitivity, durability, response, and recovery time.

No. Modes	Carbon Materials	Modes	Fabrication	Mechanism	Working Range	Sensitivity	Response/ Recovery Time (s)	Durability (Cycles)	Ref
2	Monolayer graphene	Humidity	CVD and oxygen plasma etching	Capacitive	2–90%RH	17–32%/%RH	~8/~19	1000	[185]
				Resistive					
		Temperature		Current	10–90 °C	N	~4/~10	N	
	Cracked paddy-shaped MoS_2_/graphene foam/Ecoflex	Strain	Thermal CVD, dipping, and annealing	Piezoresistive	0–22%	GF = 24.1	N	N	[186]
		Pressure			0.6–25.4 kPa	3.28–6.06/kPa	N	4000	
	SWCNTs/PDMS	Pressure	Coating and molding	Capacitance	0–25 kPa	0.7/kPa	0.05/N	10,000	[187]
	rGO	Temperature	Spray-coating	Resistive	22–70 °C	0.83%/°C	0.1/N	N	
	PDMS/SWCNT composite	Pressure	Spray coating and leather mold	Piezoresistive	0–400 kPa	0.03–7.76/kPa	0.132/0.12	10,000	[188]
	PDMS/SWCNT composite with thermochromic material	Temperature		Resistive	23–90 °C	N	N	N	
	Graphene–CNT–Silicone adhesive nanocomposite	Humidity	Doctor blade and drop casting	Impedance	36–94% RH	−84.5 Ω/%RH	26/74	N	[189]
				Capacitance		1336.7 pF/%RH			
		Temperature		Impedance	37–87 °C	−19.8 Ω/°C	34/82	N	
	PU@CNT composite	Temperature	Hot pressing	Resistive	30–110 °C	−2.84 × 10^−3^/°C	N	5000	[190]
	PU dielectric	Pressure		Capacitive	0.1–50 kPa	0.0549/kPa	0.094/0.134	5000	
	GO/SWCNTs/PDMS composite	Humidity	Screen and inkjet printing Blading and doctor blade	Resistive	25–80%RH	0.137–11.145%/%RH	0.5/0.3	N	[191]
	SWCNTs/PDMS composite	Pressure		Piezoresistive	0.024–230 kPa	27.91–77.78 /kPa	0.03/0.03	6000	
	Graphene/PEDOT:PSS hydrogel	Strain	One-pot method	Resistive	1000%	8.1	0.2/N	10,000	[192]
		Temperature			7–60 °C	−7.16–−0.162%/°C	N	N	
2	CNTs sponge/PEDOT:PSS/PDMS	Pressure	Soaking and oven drying	Piezoresistive	0–40 kPa	26.8–902.2/kPa	0.063/0.071	500	[193]
		Temperature		Resistive	20–80 °C	0.84%/°C	1.1/1.5	5 days	
3	GO	Humidity	Spray coating	Capacitive	20–90%	0.0589 pF/%RH	N	N	[194]
	rGO	Temperature		Resistive	0–100 °C	−3.4 kΩ/°C	N	N	
	PDMS	Pressure	Lamination	Resistive	0–450 kPa	0.002/kPa	0.2/N	2000	
				Capacitive					
	rGO/CNCs	Compression Strain	Mixing, freezing, freeze-drying, and carbonization	Resistive	0–99%	GF = 369.4	N	10,000	[195]
		Pressure			0.00075 kPa	N	N	N	
		Bending			0.052–180°	N	N	10,000	
	Graphene-glycerol	Strain	Coating	Piezoresistive	0–1000%	GF = 45.13	0.2/0.2	10,000	[196]
		Pressure		Resistive	0–50 kPa	80%	N	N	
		Twisting			0–180°	100%	N	N	
	PDMS-coated microporous polypyrrole/graphene foam (PDMS/PPy/GF)	Pressure	CVD, electrochemical deposition, and dip-coating	Piezoresistive	0–50 kPa	2.01/kPa	0.02/N	10,000	[197]
		Temperature		Thermoelectric	25–70 °C	49.8 µV/K	1.5/8.3	N	
		Strain		Resistive	0–50%	GF = −1.38 (<10%)	1/2.5	N	
						GF = −0.09 (10–50%)			
	Carbon fibers and MWCNTs (CFs-MWCNT) composite	Temperature		Resistive	30–50 °C	1.49–2.46%/°C	N	N	[198]
		Pressure		Piezoresistive	0–60 kPa	0.91–42.5/kPa	0.1/0.1	6000	
		Bending			0–180°	95.5%/rad	N	1000	
	GO-doped-PU nanofiber membrane coated with PEDOT	Pressure	Electrospinning, in situ polymerization, low-temperature oxygen plasma	Piezoresistive	0.001–20 kPa	0.15–20.6/kPa	0.012/N	10,000	[199]
		Strain			0–550%	10.1–193.2	N	10,000	
		Flexion			1.0 cm^−1^	N	N	6000	
	CNT/PDMS composite	Pressure	Replica molding and ultraviolet-ozone exposure	Piezoresistive	0–270 kPa	6.67/kPa	0.024/0.03	10,000	[200]
		Bending			1–6.5 mm	17.7/mm	N	N	
		Tensile strain			0–50%	GF = 409	N	N	
	Nanopapillae-decorated carbon nanosheet (NP-CNS)	Humidity	Pyrolysis and screen printing	Resistive	0–96%RH	8.25	1.7/100.1	N	[201]
		Strain		Piezoresistive	0–500%	GF = 21.9–99.9	0.07/N	N	
		Pressure			0.005–0.6 kPa	N	0.032/N	N	
3	rGO/polyorganosiloxane aerogels	Temperature	Copolycondensation	Resistive	20–100 °C	50.20%	N	10,000	[202]
		Pressure			0.01–110 kPa	83.50%	N		
		Strain			0.1–80%	84%	N		
	CNC (10 mg)-CNT (30 mg) buckypaper	Strain	Mixed vacuum filtration and curing	Piezoresistive	0–100%	GF = 352,085	0.033/0.016	10,000	[203]
	Pre-stretched CNC (10 mg)-CNT (80 mg) buckypaper	Temperature	Mixed vacuum filtration, pre-stretch, and curing	Resistive	−266.15–126.85 °C	1.88%/°C	N	10	
	CNC-CNT on cellulose filter paper (1:1)	Humidity	Dripping	Resistive	10–80%RH	N	N	10	
	Graphene woven fabric (GWF)/PDMS composite	Pressure	Catalytic decomposition and dipping	Piezoresistive	0–20 kPa	0.0142/kPa	N	1000	[204]
		Strain			0–140%	GF = 582	N	N	
		Temperature		Thermoresistive	25–80 °C	0.0238/°C	N	N	
4	CB/rGO composite	Strain	Spray coating	Resistive	N	GF = 14.6 (compression)	~0.34/N	1000	[205]
						GF = 1.8 (tension)			
		Humidity			16–95%RH	2.04/%RH	~300/~80	N	
		Temperature			20–60 °C	0.6%/°C	~100/N		
		Pressure			0–250 kPa	0.09–0.59%/kPa	~0.25/N		
	CB-PDMS	Strain	Spin coating	Resistive	0–40%	GF = 81.2 (0–5%)	<0.05/N	4000	[206]
						GF = 28.5 (5–40%)			
		Pressure			0–20 kPa	4 × 10^4^%	0.1/0.1	N	
		Flexion			0–150°	N	N		
		Temperature			25–150 °C	0.515 ppm/°C	8.4/N		
	Polyaniline-coated MWCNTs	Humidity	Two-step assembly	Conductive	30–80% RH	4.80%	25/38 (Basal layer)	2500	[207]
							56/55 (double layer)		
		Pressure		Piezoresistive	0.028–100 kPa	GF = 10	0.11/0.13	10,000	
		Bending strain			0–2.7%	GF = 35.8			
		Twisting strain			0–90°	GF = 20.8			

## 4. Discussion

**Figure 3:**
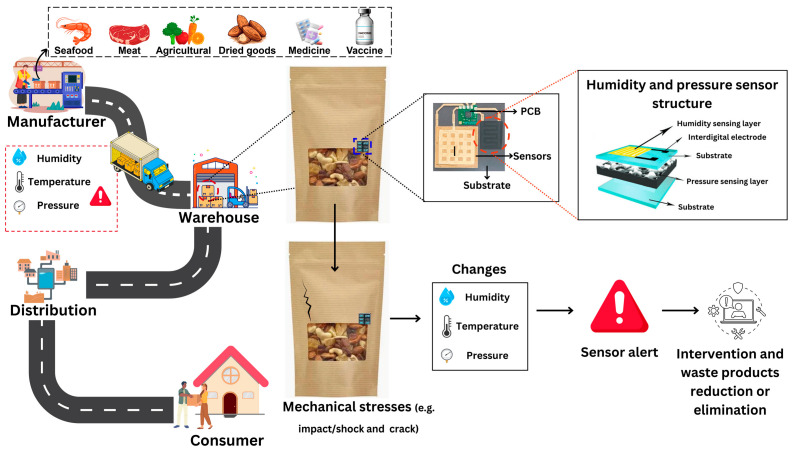
Schematic illustration of packaging with integrated carbon-based sensor systems capable of tracking humidity, temperature, pressure, and mechanical shocks in real time for monitoring food and medical products throughout transportation and storage. As the products transit from the manufacturer to the consumers, sensors continuously record environmental changes and transmit data to the centralized monitoring platforms. When deviations from set thresholds are detected, the system generates immediate alerts, enabling timely interventions and corrective actions.

**Figure 4:**
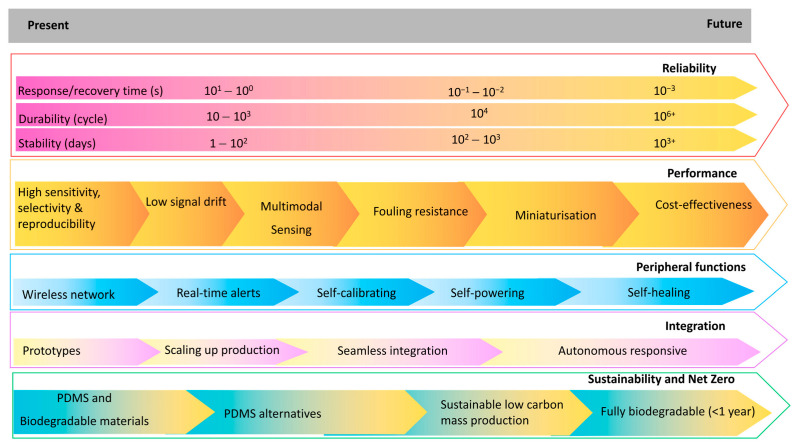
Sensors develop roadmap for smart packaging. The evolution over time is not shown to scale, and each performance and function element is not depicted as a stage of development, but rather as a feature that research is actively progressing toward. Research in the future will focus on improving sensors performance (stability, selectivity, sensitivity, reusability) and sensor integration with different sensing modalities and miniaturized size supported by IoT and AI-driven signal processing for packaging with self-healing, low energy consumption, and self-power function. Although in many publications, durability and stability are estimated by cycling tests, durability refers to the sensor’s ability to withstand physical stress, environmental conditions, and wear overtime without degrading, whereas stability is sensor’s ability to maintain consistent performance and accuracy over time and, thus, with no drift or changes in sensitivity. However, most research currently focuses on wearable applications, with dual-modal designs prevailing, as adding more sensing modes introduces challenges such as signal interference/decoupling, increased fabrication complexity, and higher costs.

There is growing research on carbon-based sensors for humidity, temperature, and mechanical stress-monitoring to improve the ability to track food and medical products during transit as illustrated by Figure 3. However, the demand for highly sensitive, durable, and scalable carbon-based sensor technologies is increasing with the global emphasis on real-time logistics monitoring. Challenges persist in translating these sensors from laboratory settings to scalable, commercially viable solutions. This systematic literature review critically assesses the recent advancements in carbon-based sensors designed to monitor humidity, temperature, and mechanical stress, either individually or as part of a multi-stimuli detection system, with potential applications in tracking food and medical products during transit. The performance and reliability of carbon-based sensors are significantly influenced by several factors, including materials selection, structural design, fabrication techniques, electrode configuration, and encapsulation strategy. These factors collectively determine the sensor’s sensitivity, responsiveness, durability, and applicability in various transportation environments. A research roadmap developed from the studies (Figure 4) shows some progress over time with efforts toward improving sensing modalities with the integration of different sensing modes as well as enhancing the functionality and properties of the sensing system, for example, with self-healing and lower power consumption or self-powered features. Alongside the need for improving the above-mentioned sensors’ performance and features, further investigation and innovations are required especially for sustainable and cost-efficient large-scale production.

The performance and reliability of carbon-based sensors is largely determined by the intrinsic properties and functional requirements of the material used. Among carbon-based materials, GO stands out for humidity sensing (Table 3) due to its low cost, large surface area and high hydrophilicity due to oxygen-containing functional groups which enhance water molecule adsorption capacity and sensitivity. However, excessive oxygen-containing groups can hinder recovery times and compromise long-term stability under high-humidity conditions [208]. Furthermore, drawbacks include potential long-term drift and low selectivity as carbon-based sensors may respond to other gases or contaminants, affecting their specificity toward water vapor.

In contrast, graphene and rGO are better suited for temperature sensors, offering exceptional electrical conductivity, thermal responsiveness, and stability. These properties enable fast response, high sensitivity, and broad detection ranges, making them suitable for most transit conditions for food and medical products including cold chain.

Mechanical sensors use the mechanical strength and piezoresistive properties of graphene for pressure sensing [170], while strain sensors benefit from the flexibility [162], conductivity, and deformation sensitivity of CNTs [173,174,176], rGO [167,177,179], and graphene [181,182]. Nonetheless, these sensors often experience structural instability and poor adhesion to substrates. The combination of GO/MWNT resulted in sensors with 1095-day stability and fast response time (0.061 s) [97]. The combination of these two materials allowed the authors to harness their complementary strengths and help offset each material’s individual limitations. Indeed graphene-based sensors typically offer faster response times and greater sensitivity but require careful structural stabilization to maintain long-term stability. In contrast, CNT-based sensors are inherently more stable, but generally exhibit slower response times. Similarly, these carbon-based materials have been used with polymers, such as PDMS [164] or PU [165], which resulted in enhanced flexibility and durability. The evolution of carbon-based sensors has increasingly shifted toward multifunctional sensing platforms, which enable simultaneous detection of multiple stimuli (e.g., pressure, strain, temperature) or analytes (e.g., gases, ions) via distinct response mechanisms, which hold significant potential for smart packaging.

Efforts to overcome these challenges have largely focused on structural design modifications and AI-driven signal processing. Multilayered architectures help minimize signal interference by physically isolating sensing components, though controlling layer thickness and interfacial properties remains a challenge. Ratiometric sensing improves accuracy by analyzing signal ratios instead of absolute values, but its reliability depends on sensor stability and calibration [209]. AI-assisted signal processing enhances detection precision through real-time filtering, noise reduction, and pattern recognition. However, integrating AI introduces challenges such as higher power consumption and computational demands, which must be addressed for practical applications. To transition from prototypes to commercial use, challenges in scalability, durability, and manufacturing must be addressed. Collaboration across materials science, engineering, and AI experts will be key to developing robust, adaptable sensors and sensing systems for real-world deployment.

**Figure 5:**
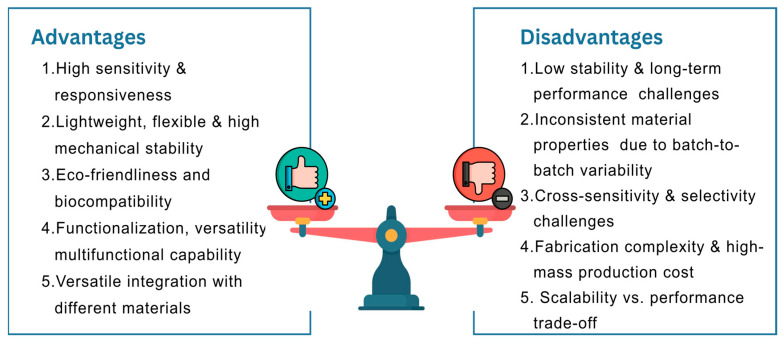
Advantages and disadvantages of carbon-based sensors.

Nano-structuring carbon materials, such as laser-induced graphene (LIG), graphene flowers, core–shell architectures, and nanoporous structures, have demonstrated significant advantages in sensor applications. The choice between hierarchical (e.g., nanostructured hybrids), hybrid (multi-material composites), or single-element systems (e.g., pure graphene) depends on the sensing targets and operational environments. These materials enhance sensitivity and response time through an increased surface area for the analyte interaction, improved electron transport at defect-engineered interfaces, good mechanical stability, and plasmonic amplification of certain carbon (e.g., graphene quantum dots, porous structure, or hybrid carbon–metal systems) via strong localized surface plasmon resonance effects, thermoplasmonic effects, and charge transfer [128]. The performance enhancements fundamentally stem from the intrinsic link between nanoscale architecture and function. For example, zero-dimensional (0D) quantum dots utilize size-dependent quantum confinement and unique optical properties, making them highly attractive for fluorescence-based sensing and plasmonic enhancement. Whereas one-dimensional (1D) and three-dimensional (3D) structures (e.g., carbon nanotubes, hierarchical porous carbons) leverage strong π–π interactions, offer interconnected diffusion pathways, and enable hierarchical analyte trapping to prevent aggregation, thereby ensuring efficient mass transport. Two-dimensional (2D) structures (e.g., LIG, graphene flowers) prioritize efficient charge transfer and expose abundant edge-reactive sites (such as dangling bonds and oxygenated defects), resulting in rapid binding kinetics. However, while these nanoscale structures provide clear advantages, they also introduce critical challenges. Particles below 20 nm often exhibit aggregation due to high surface energy, which compromises active surface accessibility and surface uniformity, ultimately affecting overall sensor performance [210]. Strategies like vertical alignment (e.g., CNT forests) or 3D carbon frameworks can partially address these challenges by spatially confining nanostructures while retaining their quantum confinement effects or plasmonic properties. Additionally, surface functionalization, template-assisted synthesis, and dispersion control strategies are actively being developed to improve uniformity and long-term stability. Although nanostructured carbon materials demonstrate excellent sensitivity and tunable electronic properties, they face challenges in processing complexity and stability compared to bulk carbon materials. Bulk carbon materials tend to provide higher mechanical integrity and stability, while nano-carbon materials are preferred for high-sensitivity and multifunctional sensing systems. From an economic perspective, the cost of carbon nanomaterials varies significantly due to production complexity, scalability, purity requirements, and applications [211,212]. GO is the most economical due to scalable synthesis, while high purity materials and CVD graphene command premium prices for specialized applications. rGO and MWCNTs offer a balance between cost and performance for conductive composites [212]. However, challenges in maintaining uniformity and quality during upscaling still exist (Figure 5). Advances in manufacturing technologies are critical to reducing costs and enhancing the scalability for widespread commercial applications. GQDs remain expensive due to low yields and niche use in biomedicine. As manufacturing techniques such as liquid-phase exfoliation, inkjet printing, and low-temperature plasma processes continue to advance, the cost of scalable materials like GO and rGO has stabilized. In the long term, the costs of high-purity carbon nanomaterials (such as CVD graphene and GQDs) are expected to decline with improved synthesis routes and increased market demand.

Single-element systems (e.g., pure graphene or CNTs) generally offer simplicity and cost-effectiveness but their limited tunability, low sensitivity and selectivity, and lack of multifunctionality restrict their use. Composites provide a promising and cost-effective approach to overcoming the limitations of single-material sensors by combining carbon materials with other (nano)materials such as polymers, metals or metal oxides, and other carbon additives. Hierarchical and hybrid systems enhance sensor capabilities by increasing surface area, controlling porosity, and optimizing molecular interactions, allowing for greater sensitivity and lower detection limits, while balancing complexity and scalability remains challenging [213]. Silicon polymers, particularly PDMS, are widely used in carbon-based sensors due to their exceptional chemical and thermal stability, biocompatibility, corrosion resistance, flexibility, and ease of fabrication [214]. These properties make PDMS an excellent choice for sensor substrates and encapsulation layers, allowing sensors to sustain large mechanical deformations while maintaining the integrity of the carbon sensing layer. Additionally, its hydrophobicity and chemical inertness protect sensitive materials from environmental factors, enhancing long-term durability. PDMS’s adaptability further enables the creation of complex structures and multifunctional sensing platforms. Despite these benefits, PDMS’s non-biodegradability poses environmental concerns, particularly in single-use applications, as its crosslinked structure makes recycling challenging [214]. Its fabrication processes also require significant energy or organic solvents [215], increasing its environmental footprint. Technically, achieving uniform carbon dispersion remains a challenge as it results in inappropriate composition, potentially reducing sensor sensitivity and reliability. Future research should explore alternative biodegradable polymers, such as PLA and cellulose derivatives to address these issues.

Recent research trends have highlighted the significant potential of hybrid composites, especially those that incorporate carbon materials with biopolymers, offering a promising balance between performance and sustainability. Cellulose and its derivates, including cellulose nanofiber (CNF) and cellulose nanocrystal (CNC), are promising candidates for enhancing the performance and sustainability of flexible carbon-based sensors. Cellulose’s renewability, biodegradability, and biocompatibility align with sustainability goals and ensure their safety for applications in food and medicine. Its abundant hydroxyl groups and water insolubility enable efficient water molecule adsorption, improving sensitivity and response times of carbon-based humidity sensors [84]. In addition to its role in composite reinforcement, cellulose exhibits a strong nanoscale and microscale response to humidity, which significantly impacts their mechanical properties. For example, a decrease in Young Modulus of cellulosic films with increasing RH values has be reported with CNC films showing smaller reduction, 15.6% (from 10.9 GPa to 9.2 GPa) when compared to other films such as xylan hemicellulose that showed 32.9% reduction (from 7.6 GPa to 5.1 GPa) for a change in relative humidity between 15% and 95% [216]. At the microscale, fiber swelling weakens inter fiber bonding, increasing porosity and reducing tensile strength and elastic modulus, leading to structural instability. While these moisture-induced effects are generally seen as mechanical weaknesses, they are cleverly exploited in paper-based humidity sensors. Humidity-induced cellulose swelling modifies the conductive network of embedded nanomaterials (e.g., carbon nanotubes, graphene oxide), altering electrical resistance or capacitance through nanoparticle separation or dielectric shifts [217]. This phenomenon forms the underpinning mechanism behind the development of sustainable, flexible, and highly responsive paper-based humidity sensors. In this regard, Khan et al., [77] produced a paper cellulose fiber/graphene oxide matrix (PCFGOM) humidity sensor with an increase in response to humidity ranging from 10% to 90% at 1 kHz and 10 kHz, respectively. Although the response time (1.2 s) and recovery time (0.8 s) were relatively good, further study is required as 24 h stability was reported. On the other hand, the mechanical stability of cellulose with carbon-based materials enhances flexibility, responsiveness, and durability of composites, as demonstrated in CNC-GO [64] and CNT-MC [148]. However, cellulose’s lower elasticity and stretchability compared to synthetic polymers like PDMS limits its application in mechanical sensors. Modifying cellulose, such as nanofibrillated cellulose [75], polydopamine-coated CNC [64], and methylcellulose (MC) [148], improve its compatibility with hydrophobic carbon materials like graphene, while increasing surface area and hydrophilicity. These modification enable uniform and strong integration with conductive carbon materials via hydrogen bonding or van der Waals interactions, enhancing load transfer and resistance to mechanical deformation and fatigue [75]. Despite these advantages, challenges such as poor sensing results, slow responsiveness, and scalability [77] limited long-term stability [106], and mechanical durability in high humidity or dynamic environments need to be addressed. Future research should focus on tailoring material concentrations, composite structures, and fabrication methods to achieve optimal performance. For example, incorporating ~30 wt% GO in CNF composites has been shown to maximize sensitivity while retaining flexibility [106]. Similarly, a CNT-to-MC ratio of 2:1 enables efficient temperature sensing without compromising mechanical flexibility [148]. Overall, these materials hold great potential for large-scale deployment in packaging sensors and transit monitoring systems, and advancing scalable manufacturing techniques such additive printing could further enhance the viability of these materials for industrial applications.

Fabrication methods significantly influence sensor performance, reproducibility, and industrial scalability. High-precision methods such as CVD produce high-quality films but are costly to consider and are relatively low profit-margin products such as food packaging. It is also energy-intensive, which together limits its large-scale industrial adoption. Solution-based methods such as coating and casting offer scalable and cost-effective alternatives but struggle with uniformity and reproducibility. Emerging approaches such as printing, additive manufacturing, and laser thermoforming enable scalable production, reducing material waste and allowing geometry customization, but require improvements in precision and production efficiency, especially material viscosity limitations for printing techniques. Achieving consistency, reproducibility, and scalability for large-scale production is a persistent bottleneck. Batch-to-batch variations and resource-intensive methods hinder industrial adoption [170]. Recent progress in machine learning-assisted fabrication and computational tools such as COMSOL Multiphysics 5.5 [74] have shown potential in optimizing spray-coating techniques and 3D-printing processes have enhanced fabrication accuracy and reproducibility, paving the way for scalable, multifunctional sensors.

Future research in carbon-based sensor technology specifically designed for food and medicine or pharmaceutical product packaging in transportation conditions should concentrate on several key areas to enhance sensor performance (stability, sensitivity, and selectivity) scalability and environmental sustainability. These include the development of scalable and eco-friendly fabrication methods, particularly for integrating biodegradable polymers such as PLA and cellulose derivatives with other carbon materials. Material concentrations should be tailored, and composite structures optimized to maximize sensor capabilities and mechanical stability. Hierarchical architectures, hybrid material combinations, and optimized single-element systems could be prioritized to address trade-offs between sensitivity, stability, and manufacturability, especially for multifunctional sensors on a single platform. Additionally, incorporating computational modeling and machine learning-driven optimization can accelerate the design of high-performance sensors. Finally, research should focus on long-term sensor stability under fluctuating humidity and mechanical strain conditions to ensure reliable real-world deployment.

Potential limitations of this review include the qualitative nature of the evaluation. A thematic synthesis based on the type of sensors and production methods was preferred as the variability in the reported approaches to sensor fabrication and the resulting performance metrics could render a meta-analysis impractical.

## 5. Conclusions

All carbon-based sensors (ACBS) for smart packaging of food and medical/pharmaceutical products are of growing interest, especially from a sustainability point of view. These sensors not only promise to enhance the safety and efficiency of supply chains but also align with increasing regulatory demands for traceability and quality assurance during transit. This review showed that humidity sensors are mainly developed with graphene oxide, whereas graphene and carbon nanotubes are predominantly used for temperature and mechanical (strains and pressure) sensors. Their performance is usually enhanced through engineering composite materials and the selection of appropriate fabrication techniques, which also determine the structural properties of the sensors. Although some progress has been made in developing all carbon-based sensors with biodegradable polymers such as cellulose, PDMS is still largely used in the reported studies, which poses environmental concerns, particularly in single-use applications. Future efforts must prioritize the development of fully biodegradable alternatives or all carbon-based sensors without PDMS with comparable properties. Cellulose and cellulose derivatives appear to be promising materials for green and sustainable sensor development and their sensitivity to moisture should be addressed. Innovations were observed in multifunctional sensor development. However, most research focused on wearable applications with dual-modal designs prevailing. This highlights clear research gaps for extending the work on wearable applications to develop sensing systems with detection ranges that meet the requirements for critical logistics scenarios like deep-frozen vaccine transport (−80 °C to −20 °C) and highly dynamic environments while ensuring scalability. Future research should focus on optimizing composite composition and structures as well as developing scalable, environmentally friendly fabrication methods to overcome current technical and commercialization barriers.

Promising prospects emerge in four key directions: (1) Sustainable hybrid systems combining biodegradable substrates such as cellulose with bio-derived conductive polymers could enable fully compostable or biodegradable sensors while maintaining performance metrics. (2) Self-healing carbon nanocomposites may revolutionize sensor durability by autonomously repairing mechanical/electrical damage during transit. (3) Integration with emerging technologies, particularly IoT-enabled blockchain tracking, AI-driven predictive analytics, and self-powered systems using triboelectric nanomaterials could transform ACBS into active components of smart logistics networks. (4) Advanced manufacturing paradigms including machine learning-assisted optimization of composite compositions and roll-to-roll manufacturing techniques may bridge the gap between lab-scale prototypes and industrial-scale production. Simultaneously, lifecycle analysis frameworks must be developed to validate the environmental benefits of ACBS against conventional electronic sensors across entire product lifetimes for packaging.

It is anticipated that cross-sector collaboration in material science, green chemistry and engineering, physics, and supply chain digitization would contribute to the next generation of ACBS to achieve parity with conventional electronic sensors within the next decade. This evolution demands a paradigm shift to embracing packaging as an active, intelligent component of food/pharma sustainable logistics ecosystems.

## Figures and Tables

**Table 1:** Sensing range and storage conditions for the safe transportation of food and medical/pharmaceutical products.

Category	Condition	Type	Range	Reference
Humidity	-	Dried food	10–65%RH	[16]
	Cold chain	Perishable food	75–95%RH	[17]
	Ambient	Pharmaceuticals	<60%RH	[18]
Temperature	Frozen	Food	−40–−18 °C	[19]
		Medical	−40–−18 °C	[19]
	Cold chain	Food	0–4 °C	[19]
		Medical	2–8 °C	[20]
	Chilled	Food	4–8 °C	[19]
		Medical	5–25 °C	[21]
	Ambient	Food	8–40 °C	[21]
		Medical	15–25 °C	[18]
Mechanical stress	Compression	Pressure	34–344 kPa	[22]
		Strain	1–15%	
	Impact/shock	Pressure	5–40 G	[23]
		Strain	1–10%	
	Vibration	Pressure	3–200 Hz	[24]
		Strain	0.1–2% over time	

**Table 2:** Inclusion and exclusion criteria for the screening iterations.

Inclusion Criteria	Exclusion Criteria
Focus on carbon-based humidity, temperature, mechanical, and multifunctional sensors for food and medical or pharmaceutical smart packaging	Review articles, conference proceedings, books, and inaccessible articles
Articles discussing sensor improvement with sufficient details on sensor design, fabrication methods, and performance metrics	Unrelated to sensor performance or improvements
English language	Purely theoretical articles
The properties suitable for transportation in Table 1.	Articles that present speculative, unvalidated, or incomplete results
Access to full text via the authors’ institution	Publication before 2013

## Data Availability

Not applicable.

## Supplementary Materials

The following supporting information can be downloaded at https://www.mdpi.com/article/10.3390/ma18081862/s1. Table S1: PRISMA Checklist. Ref. [218] is cited in the Supplementary Materials.

## Nomenclature

3D	Three-Dimensional
ACBS	All Carbon-Based Sensor
Ag	Silver
BP	Black Phosphorus
CB	Carbon Black
CDs	Carbon Dots
CFs	Carbon Fibers
CMC	Carboxymethyl Cellulose
CNC	Cellulose Nanocrystals
CNCs	Carbon Nanocoils
CNF	Carbon Nanofiber
CNHs	Carbon Nanohorns
CNS	Carbon Nanosheet
CNT−OH	Hydroxyl-functionalized Carbon Nanotubes
CNTs	Carbon Nanotubes
Co_3_O_4_	Cobalt (II,III) Oxide
CVD	Chemical Vapor Deposition
DA	Dopamine
DLS	Direct Laser-Scribed
EPD	Electrophoretic Deposition
f-rGO	Functionalized Reduced Graphene Oxide
FGS	Fragmentized rGO sponge
G	Graphene
g-C_3_N_4_	Carbon nitride
GCM	Graphene-coated microfiber
Gcvd	Graphene via Chemical Vapor Deposition
GF	Gauge Factor
GF	Graphene Foam
GNP	Graphene Nanoplatelets
GNRs	Graphene Nanoribbons
GO	Graphene Oxide
GPANI	Polyaniline/Graphene
Gpl	Plasma-Grown Graphene
GQDs	Graphene Quantum Dots
GWF	Graphene Woven Fabrics
HEC	Hydroxyethyl Cellulose
HGO	Hummer’s Graphene Oxide
HRGO	Holey-Reduced Graphene Oxide
HSMG	High Strength Metallurgical Graphene
LB	Langmuir-Blodgett
LBL	Layer-by-Layer
LDW	Laser Direct Writing
Li	Lithium
LiCl	Lithium Chloride
LIG	Laser-Induced Graphene
LiNbO_3_	Lithium Niobate
MC	Methyl Cellulose
Mg	Magnesium
Mn	Manganese
MoS_2_	Molybdenum Disulfide
MoTe_2_	Molybdenum Ditelluride
MWCNT	Multi-Walled Carbon Nanotubes
N	Nitrogen
NFC	Nanofibrillated Cellulose
NP	Nanopapillae
p-AP	p-aminophenol
P(VDF-TrFE)	Poly(Vinylidene Fluoride-Trifluoroethylene)
PAM	Polyacrylamide
PANI	Polyaniline
PCFGOM	Paper Cellulose Fiber/GO Matrix
PDA	Polydopamine
PDMS	Polydimethylsiloxane
PEDOT:PSS	Poly(3,4-ethylenedioxythiophene) Polystyrene Sulfonate
PEG	Polyethylene Glycol
PEI	Polyethyleneimine or Polyetherimide
PHEA	Poly-2-hydroxyethyl acrylate
POS	Polyorganosiloxane
PPy	Polypyrrole
PU	Polyurethane
PVA	Polyvinyl Alcohol
PVB	Polyvinyl Butyral
PVDF	Poly(vinylidene fluoride)
PVP	Poly(vinylpyrrolidone)
QCM	Quartz Crystal Microbalance
rGO	Reduced Graphene Oxide
S	Sulfur
SA	Sodium Alginate
SAO	SrAl_2_O_4_: Eu^2+^, Dy^3+^
SBS	Styrene-Butadiene-Styrene
SDBS	Sodium Dodecylbenzenesulfonate
SDC	Shellac-derived Carbon
SDS	Sodium Dodecyl Sulfate
SnO_2_	Tin(IV) Oxide
SWCNHs	Single-Walled Carbon Nanohorns
SWCNT	Single-Walled Carbon Nanotubes
TA	Tannic Acid
TDI	2,4’-Tolylene Diisocyanate
TEMPO	2,2,6,6-Tetramethylpiperidine 1-oxyl
TFBG	Tilted Fiber Bragg Grating
TFG	Tilted Fiber Grating
TOCFs	TEMPO-Oxidized Cellulose Fibers
VOCs	Volatile Organic Compounds
WO_3_	Tungsten Trioxide
ZnO	Zinc Oxide
ZrO_2_	Zirconium Dioxide
